# SpyTag/SpyCatcher Cyclization Enhances the Thermostability of Firefly Luciferase

**DOI:** 10.1371/journal.pone.0162318

**Published:** 2016-09-22

**Authors:** Meng Si, Qing Xu, Ling Jiang, He Huang

**Affiliations:** 1 College of Biotechnology and Pharmaceutical Engineering, Nanjing Tech University, Nanjing, 211816, China; 2 College of Food Sciences and Light Industry, Nanjing Tech University, Nanjing, 211816, China; 3 School of Pharmaceutical Sciences, Nanjing Tech University, Nanjing, 211816, China; 4 Department of Biochemical Engineering, School of Chemical Engineering and Technology, Tianjin University, Tianjin, 300072, China; Centro Nacional de Biotecnologia, SPAIN

## Abstract

SpyTag can spontaneously form a covalent isopeptide bond with its protein partner SpyCatcher. Firefly luciferase from *Photinus pyralis* was cyclized *in vivo* by fusing SpyCatcher at the N terminus and SpyTag at the C terminus. Circular LUC was more thermostable and alkali-tolerant than the wild type, without compromising the specific activity. Structural analysis indicated that the cyclized LUC increased the thermodynamic stability of the structure and remained more properly folded at high temperatures when compared with the wild type. We also prepared an N-terminally and C-terminally shortened form of the SpyCatcher protein and cyclization using this truncated form led to even more thermostability than the original form. Our findings suggest that cyclization with SpyTag and SpyCatcher is a promising and effective strategy to enhance thermostability of enzymes.

## Introduction

Enzyme thermostability has recently become a research focus because of its potential benefits in industrial biocatalysts. However, proteins obtained from psychrophiles usually cannot achieve their optimum catalytic activity, even at ambient temperatures. Therefore, it is desirable to obtain enzymes with a high activity at ambients or elevated temperatures is [1]. Firefly luciferase (LUC, EC 1.13.12.) catalyzes the oxidation of luciferin in the presence of Mg^2+^, ATP and molecular oxygen [2,3]. LUC is widely applied in immunoassays [4,5], biosensors [6,7], and reporter systems [8]. However, poor thermostability hampers the industrial applications of wild type LUC.

Many methods can improve the thermostability of enzymes, including directed evolution [9], rational design [10,11], saturation mutagenesis [12], chemical modification[13] and introduction of disulfide bridges [14,15]. However, these methods are time consuming and often with marginal outcomes. For example, Ma *et al*. obtained from more than 12000 mutants a thermostable mutant EAET of alkaline pectate lyases that has a 140-fold increase in the thermal inactivation (T_50_) value at 50°C; where an 84.3% decrease in catalytic efficiency was observed compared with the wild type [16]. Therefore, to meet the urgent need for enzyme stabilization, novel and efficient alternative approaches are needed.

**Fig 1:**
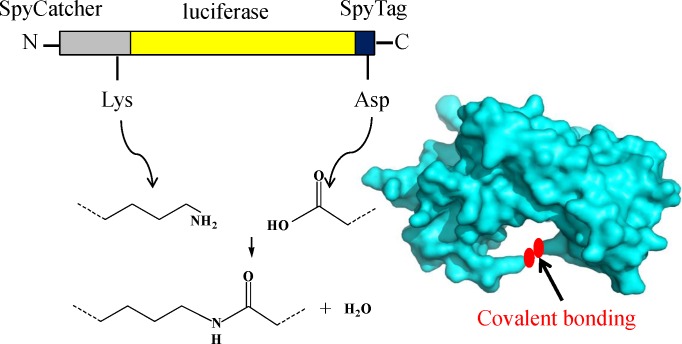
A cartoon of the SpyCatcher/SpyTag-mediated cyclization of LUC. We fused SpyCatcher and SpyTag to the N and C termini of LUC, respectively. An irreversible covalent bond formed between the reactive Lys of SpyCatcher and Asp of SpyTag during protein expression *in vivo*.

The SpyCatcher/SpyTag conjugation technique was recently discovered by splitting the isopeptide bond-forming subunit (CnaB2 domain) of *Streptococcus pyogenes* into peptide and protein fragments [17–19]: SpyCatcher (138 amino acids) and SpyTag (13 amino acids). SpyCatcher and SpyTag can rapidly conjugate covalently to form an irreversible covalent bond between the Asp^117^ of SpyTag and the Lys^31^ of SpyCatcher under nearly any common conditions (Fig 1) [20,21]. The conjugation is resistant to the force of thousands of picoNewtons and even boiling in sodium dodecyl sulfate (SDS) [22]. With the SpyCatcher/SpyTag conjugation technique, many uncommon nonlinear macromolecular topologies, including star-, tadpole-, circular- and H-shaped proteins, can be obtained [23]

In this study, we connected SpyCatcher to the N terminus and SpyTag to the C terminus of LUC, to cyclize the enzyme. The effect of this enzyme modification on LUC thermostability was then evaluated.

## Materials and Methods

### Materials

*Escherichia coli* strain BL21 (DE3) (Vazyme, China.) was used for recombinant plasmid amplification and LUC expression. Plasmid pET-22b (Novagen, USA) with a C-terminal His_6_-tag was used for gene expression. Affinity Ni-NTA spin column for His_6_-tagged proteins was obtained from Clontech (USA). DNA purification and gel extraction kits were obtained from Oxygene (Hangzhou, China). Restriction enzymes, PrimerSTAR^TM^ DNA polymerase and T4DNA ligase were acquired from Takara (Dalian, China). D-Luciferin potassium salt (LH2) was purchased from Promega (Beijing, China). All other chemicals were from Sigma–Aldrich (Shanghai, China) unless otherwise specified.

### Plasmid construction

**Fig 2:**
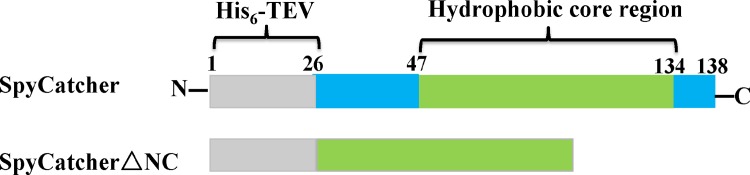
Optimization of SpyCatcher protein: a double deletion at the N-terminus (26–47 aa) and C-terminus (134–138 aa) which named SpyCatcherΔNC. All the internal regions of the SpyCatcherΔNC are identical with full-length SpyCatcher. Gray regions contain the His_6_-tag and tobacco etch virus (TEV) protease cleavage site. Blue regions indicate deleted parts in SpyCatcherΔNC.

Here, Overlap Extension PCR was used to generate pET22b SpyCatcher-LUC-SpyTag (GenBank: KX650485), pET22b SpyCatcher-LUC-SpyTagDA (reactive Asp in SpyTag converted to Ala) and pET22b SpyCatcherΔNC-LUC-SpyTag (the truncated SpyCatcher, Fig 2). We combined a fragment encoding SpyCatcher-LUC with a fragment encoding SpyTag via a 10-residue GGGGSGGGGS spacer (linker).

To add SpyCatcher or SpyCatcherΔNC to LUC via the GGGGSGGGGS spacer, the SpyCatcher-linker or SpyCatcherΔNC-linker fragment was amplified by the following primers: forward primer Sc-F1 (5’-GGAATTCCATATGTCGTACTACCATCACCATCACCATCACGATTACGACATCCCAACGACCGAAAACCTGTATTTTCAGGGCGCCATGGTT-3’) or ΔSc-F1 (5’-GGAATTCCATATGTCGTACTACCATCACCATCACCATCACGATTACGACATCCCAACGACCGAAAACCTGTATTTTCAGGGCTCAGGTGATAGTGCTAC-3’, for SpyCatcherΔNC) and reverse primer Sc-R1 (5’-ACTGCCACCGCCACCGC TACCGCCACCGCCAATATGAGCGTCACCTTTAG-3’) or ΔSc-R1 (5’-ACTGCCACCGCCACCGCTACCGCCACCGCCTTTAGTTGCTTTGCCATTTACAGTAA CCTG-3’). Next, linker-LUC was amplified from pET22b-LUC using the primers F1 (5’-GGCGGTGGCGGTAGCGGTGGCGGTGGCAGTATGGAAGACGCCAAAAACATAAA-3’) and R1 (5’-CAATTTGGACTTTCCGCCCTTCTTG-3’). The SpyCatcher-linker or SpyCatcherΔNC-linker PCR fragment was mixed at an equimolar ratio with the linker-LUC PCR fragment. The SpyCatcher-LUC or SpyCatcherΔNC–LUC fragment was amplified by the forward primer Sc-F1 or ΔSc-F1 (for SpyCatcherΔNC-LUC) and reverse primer R1. The final PCR product for SpyCatcher-LUC-SpyTag or SpyCatcherΔNC–LUC-SpyTag was amplified using the forward primer Sc-F1 or ΔSc-F1 (for SpyCatcherΔNC-LUC-SpyTag) and reverse primer Sc-R1. The final PCR product for SpyCatcher-LUC-SpyTagDA was amplified by the forward primer Sc-F1 and reverse primer ScDA-R1. The underlined letters indicate the TEV cleavage site and His_6_-tag. We digested the final PCR products with *Nde*I and *Xho*I and ligated them to the pET22b vector using T4DNA ligase, generating pET22b SpyCatcher-LUC-SpyTag, pET22b SpyCatcher-LUC-SpyTagDA and pET22b SpyCatcherΔNC-LUC-SpyTag. The insert gene was verified by DNA sequencing (Genscript, Nanjing, China).

### Protein expression and purification

The plasmids harboring the inserted gene were then transformed into *E*. *coli* (DE3) for enzyme expression. The LUC producing strain was cultured in 50 mL of Luria-Bertani (LB) medium (10% NaCl, 10% peptone, 0.5% yeast extract, and 100 μg/mL ampicillin) at 37°C and 200 rpm until the OD_600_ reached 0.8–1.0. Then, 0.1 mM isopropyl-β-d-thiogalactoside (final concentration) was added to induce protein expression at 25°C and 180 rpm for another 6 h. Cells were harvested by centrifuging the culture broth at 8000 rpm for 10 min, followed by suspending in 2 mL of 50 mM phosphate-buffered saline (PBS, pH 7.8). Suspended cells were disrupted by sonication and then centrifuged at 12,000 rpm for 10 min to remove the debris. The supernatants were loaded on Ni-NTA resin that was previously equilibrated with 50 mM phosphate buffer (pH 7.8) containing 300 mM NaCl. To remove nonspecific and unbound proteins, 50 mM phosphate buffer (pH 7.8) containing 300 mM NaCl and 30 mM imidazole was used to wash the column. LUC was eluted with 50 mM phosphate buffer (pH 7.8) containing 300 mM NaCl and 300 mM imidazole. The purified protein was analyzed by SDS-PAGE. LUC concentrations were measured via the Bradford method using the BioRad kit following the manufacturer’s instructions.

### Activity measurements

LUC activity was measured using a GloMaxTM 20/20 luminometer (Promega, USA) at room temperature (20–25°C). The assay was initiated by injecting 10 μL of enzyme solution into 100 μL of complex solution (containing 0.47 mM luciferin, 1.0 mM ATP, 10 mM MgSO_4_, 10 mM DL-dithiothreitol (DTT) and 25 mM tricine, pH 7.8). The relative light units (RLUs) of the luminometer over 10 s were used to express the enzymatic activity [24].

### Thermal stability

The half-lives (t_1/2_) of LUC were studied by incubating the purified protein solutions at 45°C in 50 mM PBS (pH 7.8) for different time intervals from 0 to 90 min, and then cooling them on ice for 10 min. The remaining activity was measured using the assay as described previously and expressed as the percentage of the original activity [24].

The T_50_ of LUC was evaluated by measuring the residual activity after incubation at various temperatures, from 25 to 55°C, for 15 min. The enzymes were placed on ice for 10 min immediately after heating, and the remaining activity was determined at room temperature.

The melting temperature (T_m_) of LUC was determined at 222 nm over the temperature range of 15 to 85°C, with a temperature increment of 1°C min^−1^ by circular dichroism (CD) measurements using a JASCO-J810 spectropolarimeter (Jasco Co., Japan) [25]. The measurements were implemented using a protein concentration of 0.1 mg/mL in 50 mM PBS (pH 7.8).

### pH stability

The purified LUC solutions (20 μg/mL) were kept in 50 mM PBS at pH range from 2 to 12 and 25°C for 30 min. The remaining activity was determined at room temperature as described above.

### pH profile and temperature optimum

The optimum pH for LUC activity was determined by injecting 10 μL (20 μg/mL) of purified enzyme into 100 μL of complex solution (containing 0.47 mM luciferin, 1.0 mM ATP, 10 mM MgSO_4_, 10 mM DTT, and 25 mM tricine) at pH range from 2 to 12. The optimum temperature of LUC was measured in the same assay buffer, pH 7.8 at the range from 10 to 80°C.

### Characterization of kinetic parameters

The Michaelis–Menten constant (K_m_) of ATP and LH2 was determined at 25°C. To estimate the Km for ATP, 50 μL of assay buffer, including 10 mM MgSO_4_, 10 mM DTT, 25 mM tricine and 2 mM LH2 (pH 7.8) was mixed with 50 μL of different concentrations of ATP (0.005–3 mM) in a 1.5-mL tube. The reaction was initiated by adding 10 μL of diluted LUC (10 μg/mL), and the relative light units of the luminometer were recorded over 10 s. The LH2 kinetic constants were determined in a similar way, but different concentrations of LH2 (0.002–2 mM) were mixed with 50 μL of assay reagent (pH 7.8), including 10 mM MgSO_4_, 10 mM DTT, 25 mM tricine, and 2 mM ATP. Apparent kinetic parameters were calculated from Lineweaver–Burk plots using Origin 8.0 software (Origin Lab, USA).

### Bioluminescence emission spectra

Bioluminescence emission spectra were recorded using a Hitachi: F-2500 Fluorescence Spectrophotometer from 400 to 700 nm. First, 500 μL of each purified LUC solution (50 μg) was added to 1.5 mL of assay buffer containing 0.47 mM luciferin, 1.0 mM ATP, 10 mM MgSO_4_, 10 mM DTT and 25 mM tricine (pH 7.8 and 5.5, respectively). Next, the scan rate, slit width, detector voltage, scan speed, and response time were controlled to optimize instrument response [26]. No remarkable loss of light was observed during each measurement.

### CD spectra

CD spectra were recorded on a JASCO-J810 spectrofluorometer. Far-UV (190–260 nm) CD spectra were collected at 0.1 mg/mL LUC in 50 mM PBS (pH 7.8) at 25°C and 40°C, respectively, using a 1 mm path length cell [26]. The JASCO-J810 software was used to smoothen the noise in the data. The resulting spectra are the average of three scans. The molar ellipticity was determined [θ] = (θ × 100 MRW) / (c × l) where θ is the measured ellipticity in degrees at a given wavelength, c is the protein concentration in milligrams per milliliter, and l is the light path length in centimeters [27].

### Intrinsic fluorescence spectroscopy

The purified enzymes were dialyzed in 100 mM PBS (pH 7.8). Fluorescence studies were conducted using a spectrofluorometer (Perkin-Elmer LS55, USA). The intrinsic fluorescence was determined using 20 μg/mL LUC solutions, and the emissions were scanned between 300 and 450 nm at an excitation wavelength of 270 nm. All spectra were collected at 25°C. The resulting spectra were corrected by subtracting the corresponding blank samples without protein.

## Results

### Expression and purification of cyclized LUC

**Fig 3:**
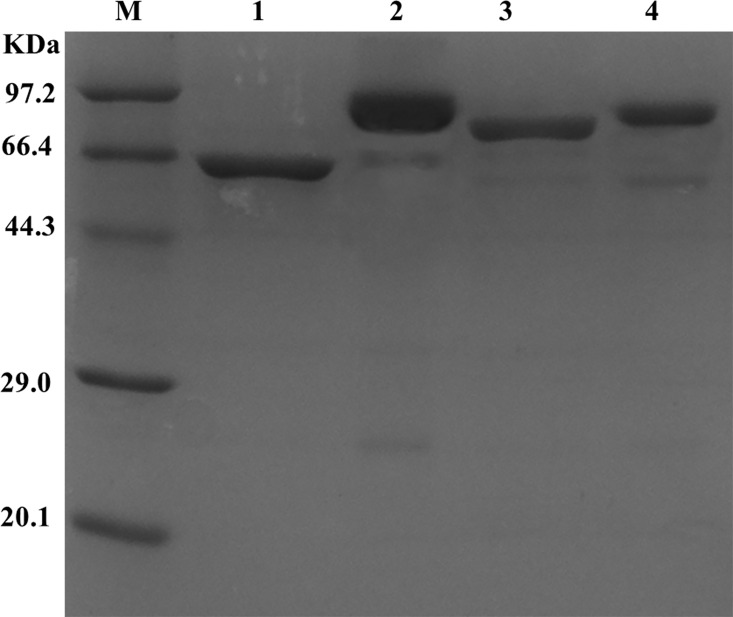
SDS-PAGE of the purified LUCs. Lane M is the protein molecular weight markers. Lane 1, 2, 3, 4 are LUC, circular LUC, linear LUC and truncated circular LUC, respectively, purified using Ni-NTA affinity chromatography.

SpyCatcher and SpyTag were genetically fused to the N and C termini of LUC, respectively, to generate cyclized LUC (SpyCatcher-LUC-SpyTag). To generate a control with linear structure, the reactive Asp in SpyTag was mutated to Ala (SpyCatcher-LUC-SpyTagDA) [28]. As a result, SpyCatcher and SpyTag could not conjugate covalently (the linear protein in this paper refers to SpyCatcher-LUC-SpyTagDA unless otherwise specified). SDS-PAGE was used to analyze the linear mutant and cyclized construct. Fig 3 shows that the purified wild type LUC (lane 1) exhibited a band of approximately 61 kDa, and the cyclized LUC exhibited a band of 80 kDa. The linear LUC exhibited faster mobility than the circular form[28]. This finding was in accordance with efficient cyclization.

### Thermostability of cyclized enzyme

**Fig 4:**
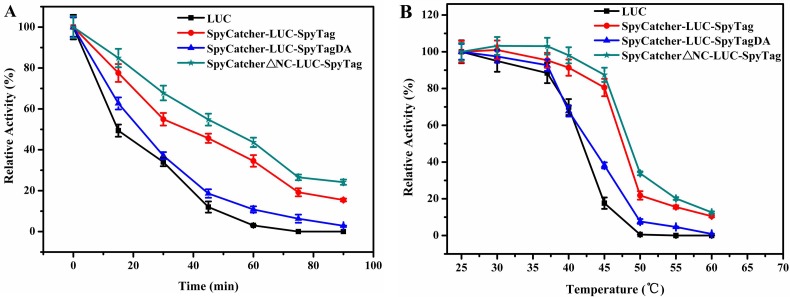
The thermostabilities of the LUCs. **(A)** The residual activities of the enzyme were measured after incubation at 45°C for different time intervals from 0 to 90 min **(B)** The residual activities of the enzyme were measured after incubation at various temperatures, from 25 to 55°C, for 15 min. Experiments were performed in triplicate and error bars correspond to the standard deviation.

To test whether cyclization protein had improved enzyme performance, the purified enzymes were subjected to thermostability tests. Fig 4A shows that the cyclized LUC was more resistant toward thermal inactivation and still maintained 35.6% of its original activity after incubation at 45°C for 60 min, whereas the wild type had lost nearly all of its activity. The half-lives (t_1/2_) of cyclized LUC was also more than twice that of the wild type.

We incubated cyclized and wild type LUC in PBS (pH 7.8) over a wide range of temperatures (Fig 4B) to determine the T_50_ values of LUC. The experimental results showed an obvious downward trend of the wild type when the temperature reached 45°C. By contrast, the cyclized LUC remained stable at this temperature. The T_50_ for cyclized LUC was 47.5°C, which was increased by 10.1°C compared with that of the wild type.

We also tested the T_m_ using CD spectroscopy to characterize the thermal effect on the conformational stability of the cyclized protein. As expected, the T_m_ value of cyclized LUC (70.2°C) exhibited significant enhancement compared with that of the wild type (53.5°C) (Table 1). This result indicated that the cyclized-LUC protein showed improved thermal stability compared with that of the wild type LUC.

**Table 1:** Kinetic properties of LUCs. Values are means ± S.D. for three independent measurements.

Enzyme	Relative activity^a^ (%)	Km (μM)		t_1/2_^b^ (min)	T_50_^c^ (°C)	Tm^d^(°C)	Optimal temp. (°C)	Optimal pH
		ATP	LH2					
**Wild type**	**100±2.4**	**105±3.2**	**16±1.3**	**16.7**	**37.4**	**53.5**	**25**	**8**
**Cyclized LUC**	**103±2.1**	**105±2.4**	**15±0.8**	**40.3**	**47.5**	**70.2**	**35**	**9**
**Truncated form**	**103±3.3**	**105±3.0**	**15±0.9**	**51.4**	**49.2**	**73.1**	**35**	**9**

^a^ Specific activity measurements were carried out at room temperature as described in Materials and methods.

^b^ t_1/2_ is the half-life at 45°C.

^c^ T_50_ is temperature at which LUCs lose 50% activity during 15 min of incubation.

^d^ T_m_ is melting temperature of LUCs.

Fig 4A and 4B show that the thermostability of LUC was in the following order: circular protein > linear form > wild type. The results indicated that the SpyCatcher/SpyTag protein fused to the termini of LUC affected the thermostability of the protein, but the improvement in the thermostability was caused mainly by protein cyclization.

### pH stability

**Fig 5:**
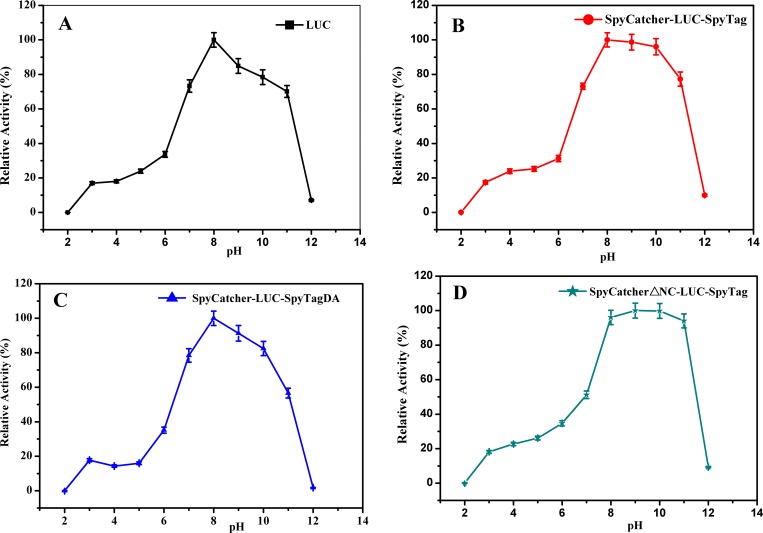
The pH stability of the LUCs. The LUCs were incubated in various buffers, at the pH range of 2.0–12.0 for 30 min at 25°C and then were assayed for residual activity at room temperature. Experiments were performed in triplicate and error bars correspond to the standard deviation.

As shown in Fig 5B, circular LUC was stable at a pH range of 8.0–10.0, maintaining nearly 100% activity after incubation in various pH buffers for 30 min. However, the wild type and linear LUCs maintained only 80% of their original activity after incubating at pH 10.0 for 30 min (Fig 5A and 5C). This result suggested that the enhancement of pH stability was a result of the cyclization of the protein.

### Effect of pH and temperature on the activity of LUCs

**Fig 6:**
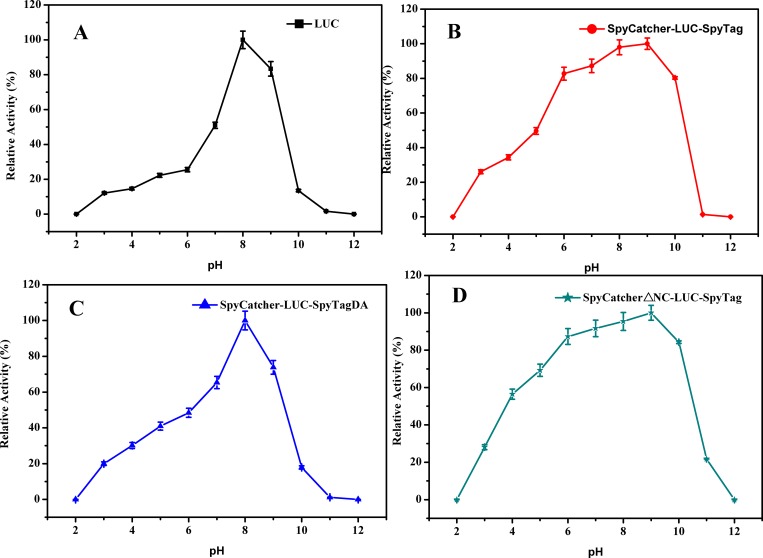
Optimal pH values of the LUCs. The activity was determined by injecting purified enzyme into assay buffers with different pHs (pH 2.0–12.0). Experiments were performed in triplicate and error bars correspond to the standard deviation.

**Fig 7:**
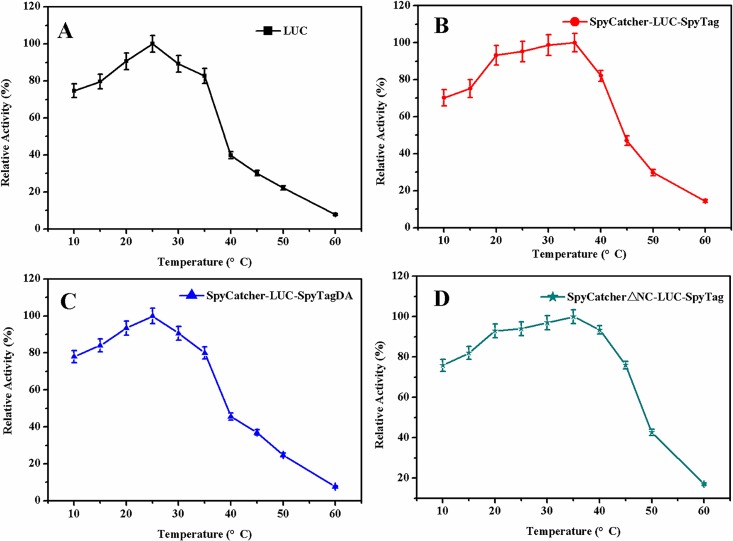
Optimal temperature of the LUCs. The enzyme activity at a temperature gradient ranging from 10 to 60°C was determined. Experiments were performed in triplicate and error bars correspond to the standard deviation.

To determine the effect of pH, the activities of the LUCs were measured over a pH range of 2.0–12.0 at 25°C. The optimum pH of circular LUC was found to be 9.0 (Fig 6B), which was 1 unit higher than that of the wild type and linear LUCs (Fig 6A and 6C). This result indicated that the circular LUC was alkali-tolerant compared with the wild type and linear forms. Similarly, the activities of the LUCs were determined at various temperatures, from 10 to 60°C. As shown in Fig 7B, the optimum reaction temperature of circular LUC was 35°C, which was increased by 10°C compared with wild type and linear LUCs (Fig 7A and 7C). In addition, circular LUC retained high activity over a broad range of temperatures, 20–40°C, whereas, wild type and linear LUCs lost most of their catalytic activity at 40°C. This result indicated that the cyclization improved the thermostability of LUC, a finding in accordance with the experimental results of T_50_ and T_1/2._

### Kinetic constants of LUCs

To detect the effects of cyclized protein on catalytic activity, Lineweaver–Burk plots were applied to calculate the apparent kinetic parameters for LH2 and ATP. As shown in Table 1, the Km values of cyclized LUC for ATP and LH2 were 105 and 15, respectively, which was almost the same as that of wild type (105 and 16, respectively). This result indicated that cyclized LUC-based SpyCatcher/SpyTag chemistry can improve the thermostability of the enzyme without impairing the biocatalytic activity.

### Structural analysis

**Fig 8:**
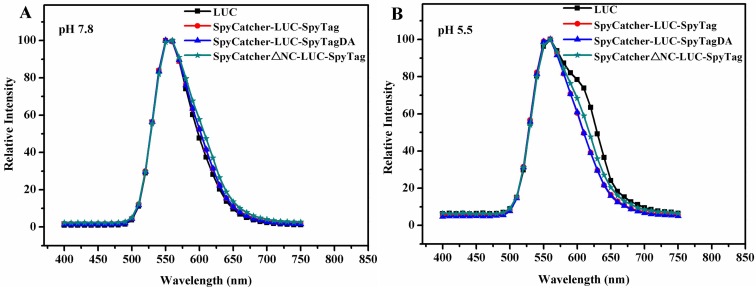
**Bioluminescence emission spectra (400–750 nm) produced by the LUCs at 25°C (A) pH 5.5 and (B) pH 7.8, respectively.** Enzyme (50 μg) was assayed with 1.5 ml mix buffer containing 0.47 mM luciferin, 1.0 mM ATP, 10 mM MgSO_4_, 10 mM DTT and 25 mM tricine, adjusted to pH 7.8 and 5.5, respectively.

Protein modification might result in the red or blue shift in the emitted color [29]. The *in vitro* bioluminescence spectra of wild type and circular LUC at pH 5.5 and pH 7.8, respectively, were obtained (25°C). As depicted in Fig 8A and 8B, although the shape of wild type LUC was slightly changed under acidic conditions, the wild type and circular LUC exhibited a similar spectrum with only a peak at 555 nm both at pH 5.5 and pH 7.8, indicating that protein cyclization did not have any significant effect on the mechanism of color emission [30]. Therefore, protein cyclization using SpyCatcher/SpyTag not only keep the basic and critical residues of LUC unchanged [31,32] but also improved the stability without hampering the catalytic activity of the protein. This observation was consistent with our enzyme activity test in that the relative activity of cyclized LUC was 103%, which was nearly the same as the wild type (100%) (Table 1).

**Fig 9:**
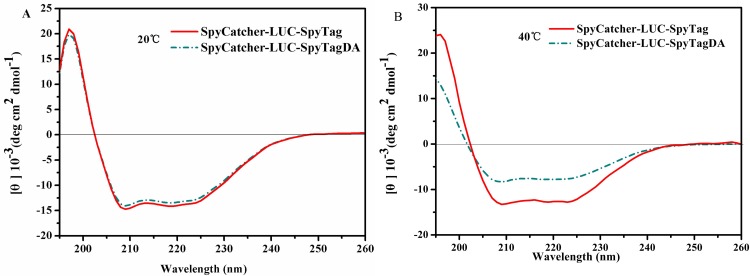
CD spectra (190–260 nm) of linear and circular LUCs. CD spectra were collected at 0.1 mg/mL LUC in 50 mM PBS (pH 7.8) at (A) 25°C and (B) 40°C, respectively.

To further understand the effect of cyclization on protein structure, purified linear and circular LUC were compared further through CD spectroscopy. As indicated in Fig 9A, the CD spectra of the linear and circular LUC exhibited a similar distribution of secondary structure when the LUC strain was grown at 25°C, indicating that cyclized LUC can fold properly like wild type. Furthermore, a noticeable difference in the conformation of circular LUC was observed after growing at 40°C (Fig 9B). The signal of the CD spectrum of circular LUC was higher than that of the linear form, indicating that cyclization makes the structure of enzyme more stable and expectedly promotes better folding after incubation at higher temperature.

**Fig 10:**
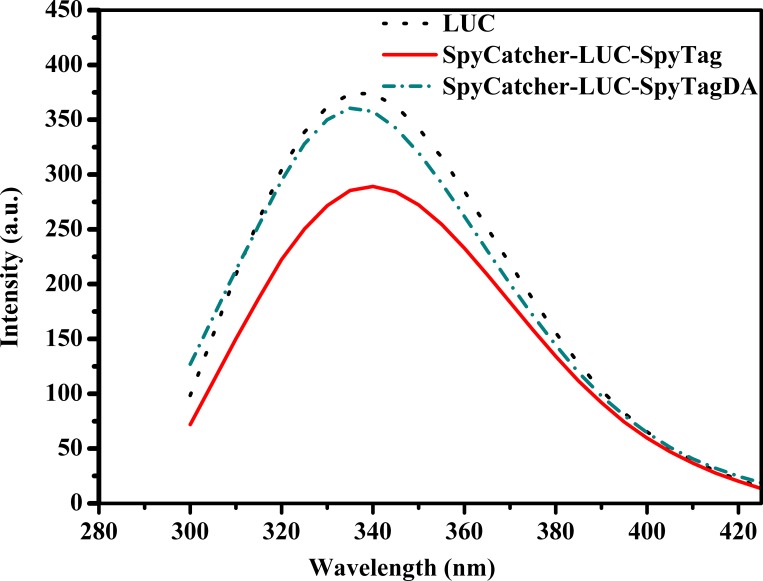
Fluorescence spectra of the LUCs. Spectra were recorded at 25°C in 50 mM PBS (pH 7.8) using 20 μg/mL LUC solution. The excitation wavelength was 270 nm.

On the other hand, there are two tryptophans, Trp 417 and Trp 426, in P*py* LUC, which are located near the protein surface of the N-terminal domain [30]. The intrinsic fluorescence of tryptophans is highly sensitive to the local microenvironment. The variation in the microenvironment of tryptophans may lead to conformational changes. As shown in Fig 10, no significant differences in the fluorescence spectroscopy of linear and wild type LUCs were observed. In addition, an evident decrease in fluorescence intensity was observed in the cyclized LUC, indicating that the cyclization of LUC altered the microenvironment polarity of the tryptophan residue (417) to a more hydrophilic environment[33]. This could explain the improvement in the thermostability of circular LUC.

### Optimization of SpyCatcher-LUC protein

Structural analysis revealed that not every amino acid in SpyCatcher is essential for conjugating with SpyTag. As described by Howarth and coworkers’ study, aa 53–118 of SpyCatcher are necessary to conjugate covalently. Liu ZD [34] and Howarth [35] found that full-length and truncated SpyCatcher protein can react with SpyTag efficiently. To investigate the effects of these non essential amino acids on the thermostability of circular LUC, a double deletion of SpyCatcher at the N terminus (26–47 aa) and C terminus (134–138 aa), called SpyCatcherΔNC, was fused to the N terminus of LUC, with the SpyTag fused to the C terminus (Fig 2). As a result, a new cyclized protein called SpyCatcherΔNC-LUC-SpyTag was generated. The thermostability of the original and truncated cyclized proteins were compared. The t_1/2_,T_50_ and Tm values for truncated cyclized LUC were 51.4 min, 49.2°C and 73.1°C, respectively, which were higher than those for the original cyclized protein (40.3 min, 47.5°C and 70.2°C, respectively). The truncated cyclized LUC was more resistant to alkaline conditions because it maintained nearly 100% of its activity after maintaining pH 8.0–11.0 for 30 min (Fig 5D). However, original cyclized LUC remained only 75% activity of its original activity after incubating at pH 11.0 (Fig 5B). In addition, catalytic activity of the truncated cyclized protein kept unchanged compared with the original circular form. These observations indicated that truncation of SpyCatcher was important in improving stability of cyclized LUC.

## Discussion

In summary, we successfully improved the stability of LUC through SpyCatcher-SpyTag chemistry without hampering its catalytic activity. Cyclized LUC can be expressed at a high cell density, and the yield of protein can reach 16–18 mg/L in shake flask fermentation, similar to that of wild type LUC. Compared with the wild type LUC, the t_1/2_, T_50_ and T_m_ of cyclized protein were increased by 2.41 times, 10.1°C and 16.7°C, respectively. The cyclized LUC showed an optimum temperature of 35 and an optimum pH of 9.0 and maintained nearly 100% activity after incubation at pH 10.0 for 30 min, indicating that cyclized LUC can resist the alkaline condition. The results were consistent with those of a previous study on SpyCatcher/SpyTag-mediated cyclization by Wang JD [36]. They dramatically improved the thermostability of lichenase by SpyCatcher/SpyTag-mediated spontaneous cyclization. From the structural analyses, we can conclude that the basic and critical residues for cyclized LUC remain unchanged. Moreover, cyclization did not change the secondary structure of the protein, and cyclization can promote better folding after growth at high temperature. The mechanisms involved in thermostability enhancement can be attributed to a more stable structure and the change in the conformation of the cyclized LUC compared with that of the wild type. In addition, we found that the truncated SpyCatcher was beneficial to improving the thermostability of cyclized LUC.

Many reports have shown that the thermostability of LUC can be improved by site-directed mutagenesis [37,38]. However, most site-mutagenesis methods are based on information about the protein structure and are time consuming. For example, Koksharo et al. [39] obtained a thermostable mutant 4TS from four cycles of random mutagenesis and the screening of approximately 1000 colonies in a typical random mutagenesis cycle, which produced one or two different thermostable mutants. Surprisingly, no structural information was required when using SpyCatcher/SpyTag chemistry, broadening the application of the conjugation technique.

It has been reported that the cyclization of protein can be achieved using protein labels such as inteins[40–42], sortases [43] and carbodiimide cross-linking [44]. However, some disadvantages usually limit the application of these protein labels. For example, sortase A needs to be limited at the chain termini of the protein to perform ligations [43]. For intein-based cyclization, the obligatory fusion with the target protein can hamper the catalytic activity of the protein in some situations [32]. The range of inteins for circularization is restricted because high-affinity intein is needed to isolate the circular product. Compared with the common protein labels, SpyCatcher/SpyTag chemistry is reactive at the terminal and internal sites of a protein[45] and can improve the stability of protein without changing its function. Moreover, the purification of the tagged protein is simple using the SpyCatcher/SpyTag conjugation technique because the interaction between SpyCatcher and SpyTag is irreversible.

Research has shown that a decrease in reconstitution can be observed between the truncated SpyCatcher and SpyTag *in vitro* [35]. In our study, we found fused truncated SpyCatcher and SpyTag at the N and C termini of LUC, respectively, and they were expressed *in vivo*. Interestingly, the truncated SpyCatcher can react with SpyTag as effectively as the original SpyCatcher and exhibit higher thermostability than the original cyclized LUC. Further work may optimize the SpyCatcher protein to obtain higher thermostability of cyclized proteins and broaden the industrial application of enzymes.
